# (+)-Podocarpic Acid as Chiral Template in the Synthesis of Aphidicolane, Stemodane and Stemarane Diterpenoids †

**DOI:** 10.3390/molecules21091197

**Published:** 2016-09-08

**Authors:** Angela La Bella, Francesca Leonelli, Luisa Maria Migneco, Rinaldo Marini Bettolo

**Affiliations:** 1 Dipartimento di Chimica, Università degli Studi di Roma “La Sapienza”, P.le Aldo Moro, 5, I-00185 Roma, Italy; angela.labella@uniroma1.it (A.L.B.); luisamaria.migneco@uniroma1.it (L.M.M.); 2 Dipartimento di Biologia Ambientale, Università degli Studi di Roma “La Sapienza”, P.le Aldo Moro, 5, I-00185 Roma, Italy; francesca.leonelli@uniroma1.it

**Keywords:** aphidicolin, stemodin, stemarin, podocarpic acid, synthesis

## Abstract

In this review the synthetic work in the field of aphidicolane, stemodane and stemarane diterpenoids, in which readily available (+)-podocarpic acid (**4**) was used as chiral template for the construction of their polycyclic structures, is described as it developed along the years. In the frame of this work (+)-podocarpic acid (**4**) was a very useful tool in a model study leading to the syntheses of tetracyclic ketones **7** and **8**, models of key intermediates **5a** and **6** in the syntheses of (+)-aphidicolin (**1**) and (+)-stemodin (**2a**), respectively. (+)-Podocarpic acid (**4**) was also converted into (+)-2-deoxystemodinone (**2d**), allowing confirmation of the stemodane diterpenoids absolute configuration, into (+)-aphidicol-15-ene (**36**) and into *Stemodia chilensis* tetracyclic diterpenoid (+)-19-acetoxystemodan-12-ol (**2f**), allowing confirmation of its structure. (+)-Podocarpic acid (**4**) was then extensively used in the work which led to the synthesis of (+)-stemar-13-ene (**57**) and (+)-18-deoxystemarin (**3b**). Finally, (+)-**4** was converted into (+)-2-deoxyoryzalexin S (**66**), which made it possible to demonstrate that the structure of (+)-**66** could not be attributed to a Chilean *Calceolaria* isolated diterpenoid to which this structure had been assigned.

## 1. Aphidicolane, Stemodane and Stemarane Diterpenoids

**Figure 1:**
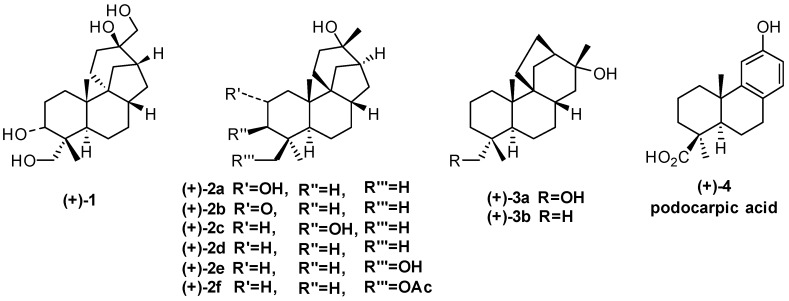
Aphidicolin **1**, stemodane diterpenoids **2**, stemarane diterpenoids **3** and podocarpic acid **4**.

Between 1970 and 1980 three new classes of tetracyclic diterpenes, whose C/D ring system is constituted by a bicyclo[3.2.1]octane system, were isolated. The parent compounds of these classes are: (+)-aphidicolin (**1**), (+)-stemodin (**2**) and (+)-stemarin (**3a**) (Figure 1). (+)-Aphidicolin (**1**), which showed interesting biological properties, was isolated in 1972 from the fungus *Cephalosporium aphidicola* Petch [1,2]. (+)-Stemodin (**2a**) was isolated in 1973, along with (+)-stemodinone (**2b**), from *Stemodia maritima* L. [3]. In 1975 (+)-stemarin (**3a**) [4] and stemodane diterpenoid maritimol (**2c**) [5] were also isolated from the same plant. The number of isolated diterpenoids of these classes grew in the following years. After the disclosure of these new and interesting structures various research groups over the world decided to engage themselves in the synthesis of these tetracyclic diterpenoids. Some among these groups decided to enter the field starting from the preformed system of (+)-podocarpic acid (**4**) (Figure 1) [6]. Hereafter the work of the latter groups is described the way it developed along the years.

**Figure 2:**
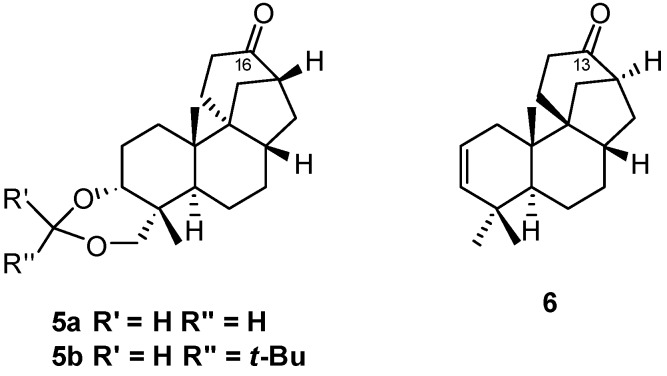
Key synthetic intermediates in the synthesis of **1** and **2a**, **2b**, **2d**.

The first two formal total syntheses of (±)-aphidicolin (**1**) were published at the beginning of 1979 [7,8]. Both syntheses had as a target compound **5a**, the acetonide of the periodic acid cleavage product of (+)-**1**, reconverted, though not diastereoselectively into (+)-**1** [2]. This synthetic problem was overcome in 1988 when a solution to it was described [9,10]. Later syntheses of (±)-**1** via **5a** and **5b** (Figure 2) were published also by other groups [11,12,13,14,15,16,17,18,19]. Enantioselective total syntheses of (+)-**1** via **5a** and **5b** were also described [20,21,22]. In 1980 the first total synthesis of (±)-stemodin **2a** and (±)-stemodinone **2b** via the tetracyclic ketone **6** (Figure 2) was also published [23].

## 2. 1982 Synthesis of 17-Noraphidicolan-16-one and 17-Norstemodan-16-one from (+)-Podocarpic Acid via (−)-9(11)-Podocarpen-12-one (1982)

Between 1976 and 1978 one of us (RMB) had been involved, as a member of the Wiesner group at the University of New Brunswick (Fredericton, NB, Canada), in the synthesis of some diterpene alkaloids whose C/D ring system, constituted by a bicyclo[3.2.1]octane system, was obtained by the bicyclo[2.2.2]octane → bicyclo[3.2.1]octane skeletal rearrangement [24,25,26]. The end of this fruitful and exciting experience coincided with the disclosure of the first two syntheses of (±)-aphidicolin (**1**) [7,8]. Thus, back in Rome, we desired to show that the approach developed by Wiesner for the construction of the C/D ring system of diterpene alkaloids [27,28,29,30] could be also particularly convenient for the synthesis of the aphidicolane and stemodane systems. In fact the above rearrangement would have not only produced the required bicyclo[3.2.1]octane system but also introduced the oxygenated function present at C-16 in **5a** and at C-13 in **6**. Our initial aim was, therefore, that of showing the usefulness of this strategy by obtaining the tetracyclic ketones **7** and **8** which differ from **5a** and **6** only in the A ring substitution, respectively. This choice would permit adoption of (+)-podocarpic acid (**4**) as starting material.

### 2.1. Retrosynthetic Analysis and Strategy

The approach adopted by us for obtaining **7** and **8** was based on the following retrosynthetic analysis: (a) obtaining the bicyclo[3.2.1]octane system by rearrangement of a bicyclo[2.2.2]octane intermediate [31,32,33,34]; (b) obtaining the bicyclo[2.2.2]octane intermediate by intramolecular aldol reaction [35,36,37]; (c) stereoselective addition of a –CH_2_CHO unit or synthetic equivalent from the α face to the C(9) of (−)-9(11)-podocarpen-12-one intermediate **10a**. For the sake of precision the synthesis of (±)-**1** described in ref. [12] also proceeds via a 9(11)-podocarpen-12-one and is also based on the bicyclo[2.2.2]octane → bicyclo[3.2.1]octane skeletal rearrangement; in this synthesis the bicyclo[2.2.2]octane system is obtained by Diels-Alder addition. Previously a synthesis of (±)-**2c** based on the same approach was also described [38].

**Scheme 1:**
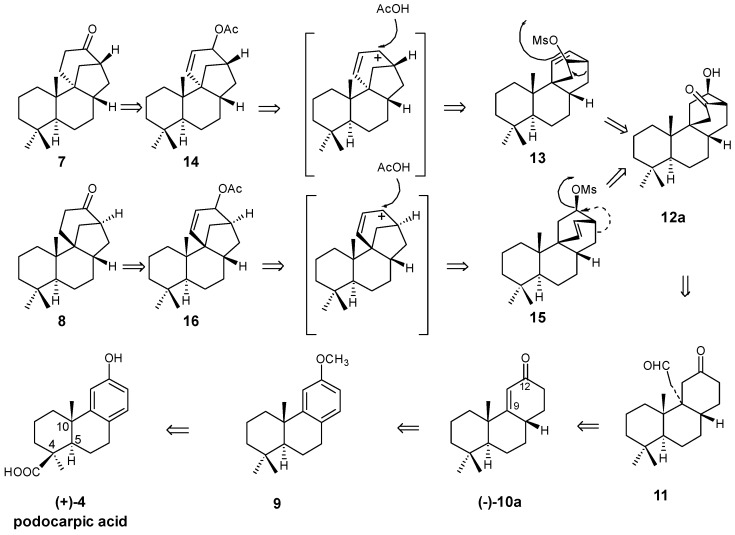
Retrosynthetic analysis of the preparation of tetracyclic ketones **7** and **8**.

As it can be observed (Scheme 1) the migrating bond is always the same one. Nevertheless, the different location of the leaving group causes its migration via the upper or lower face, leading to the stemodane and aphidicolane systems, respectively.

### 2.2. (+)-Podocarpic Acid as Chiral Template and (−)-9(11)Podocarpen-12-one as Suitable Chiron for Obtaining of 17-Noraphidicolan-16-one and 17-Norstemodan-16-one

The commercially available (Aldrich) chiral template [39] (+)-podocarpic acid (**4**) appeared to us a suitable starting material for a quick evaluation of our working hypothesis. In fact, it shows a good overlap of the skeleton and correctly set-up stereogenic centers at C(5) and C(10).

**Scheme 2:**
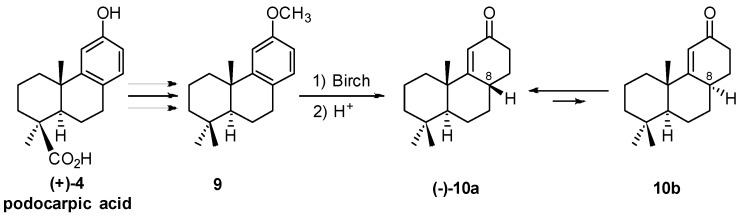
Birch reduction of podocarpic acid derivative **9** to give after acidic hydrolysis (−)-9(11)podocarpen-12-one (**10a**).

Besides, it was also known that the Birch reduction of **9**, available in few steps from (+)-podocarpic acid (**4**), followed by acidic cleavage of the resulting dienol ether gives the *chiron* [39] (−)-9(11)podocarpen-12-one (**10a**) whose vinylogous H-C(8) is β-oriented as required [40] (Scheme 2). This is because the structure **10a** is thermodynamically more stable than the epimeric one **10b** in which the H-C(8) is α oriented [41].

Therefore the use of (+)-podocarpic acid (**4**) as starting material would have also resulted in the correct installment of the stereocenter at C(8) and generate a handle (the ring C α,β-unsaturated ketone) for the construction of the bicyclo[2.2.2]octane intermediate by the Wiesner two-carbon annulation sequence [42].

### 2.3. Synthesis of 17-Noraphidicolan-16-one (1982)

**Scheme 3:**
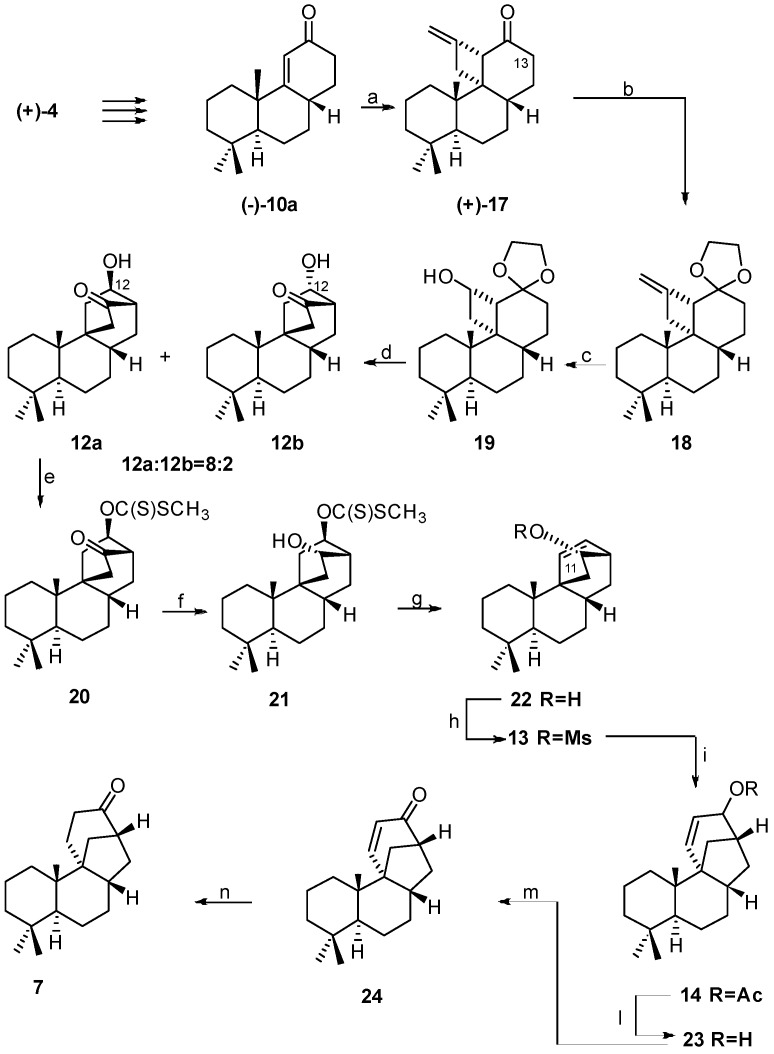
Synthesis of 17-Noraphidicolan-16-one. *Reagents and conditions*: (**a**) allene, hν, pyrex, −78 °C, THF, quantitative yield; (**b**) ethylene glycol, TsOH, benzene, reflux; (**c**) O_3_, EtOH/CH_2_Cl_2_ = 1:1, −78 °C/NaBH_4_; (**d**) (1) THF/1N HCl = 2:1; (2) MeOH/1N NaOH; 70% from **18** (**e**) NaH, CS_2_, CH_3_I, THF, reflux, 80%; (**f**) NaBH_4_ MeOH/EtOH = 1/1, −10 °C; (**g**) *o*-xylene, 190 °C, 4 h, 90%; (**h**) MsCl/Py, 0 °C, 95%; (**i**) AcOH, 50 °C, 95%; (**l**) 5% KOH (MeOH); (**m**) PCC/Al_2_O_3_, 80% from **14**; (**n**) H_2_/10% Pd(C), 95%.

The starting material for the synthesis of **7** (Scheme 3) was (−)-9(11)-podocarpen-12-one (**10a**). Allene photoaddition to **10a** in THF at −78 °C gave the photoadduct (+)-**17**. The stereochemistry of the addition was hypothesized on the basis of the Wiesner empirical rule [43,44,45,46] and later confirmed by the preparation of the expected final product. Nowadays the regio- and stereochemistry of the allene photoaddition can be established by 2D-NMR experiments (see Section 7). The exocyclic methylene in acetal **18**, obtained from (+)-**17** by standard methods, was then converted into the cyclobutanol **19** by oxidative cleavage followed by reduction. Treatment of the latter with diluted HCl unmasked the carbonyl function and effected cyclobutanol ring opening by a retro-aldol reaction (see Section 8.1). Subsequent diluted NaOH treatment in MeOH afforded **12** as a mixture of epimers at C(12). In order to obtain the aphidicolane system three synthetic operations were required:

- protection of the HO-C(12);
- reduction of the carbonyl group to the corresponding 15α-alcohol;
- introduction of a double bond at C(11) in order to make the rearrangement quantitative.

This was achieved by transforming the HO-C(12) into the corresponding dithiocarbonate **20**. Subsequent reduction of the carbonyl group with NaBH_4_ gave stereoselectively the desired alcohol **21** owing to the bulkiness of the dithiocarbonate group. The following Chugaev reaction gave finally the unsaturated alcohol **22** which was then transformed into the methanesulfonate **13**. The latter, submitted to solvolysis in glacial acetic acid, gave the rearranged acetate **14** (see Section 8.2). Saponification of the latter followed by oxidation with PCC/Al_2_O_3_ afforded the enone **24** which was eventually reduced to the target ketone **7** [47], identical with an authentic sample kindly donated by Dr. B. Hesp (ICI Americas Inc., Wilmington, DE, USA) and Dr. A.H. Ratcliffe (ICI Ltd. Macclesfield, UK).

### 2.4. Synthesis of 17-Norstemodan-16-one (1982)

**Scheme 4:**
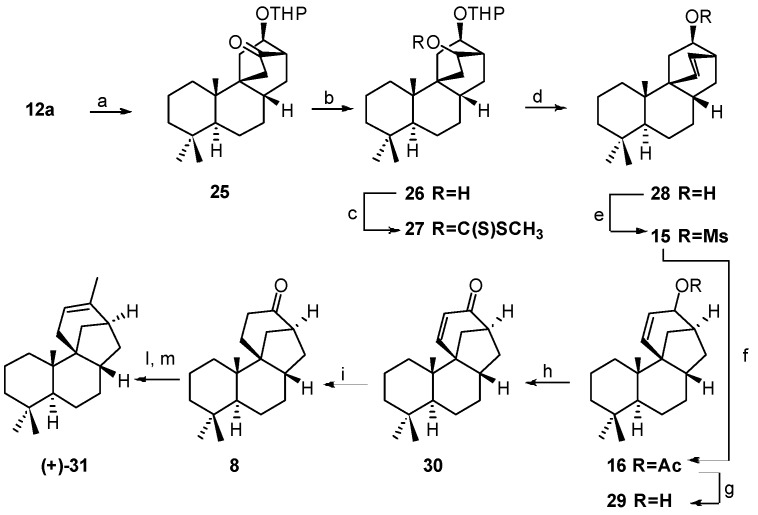
Synthesis of 17-Norstemodan-16-one. *Reagents and conditions*: (**a**) DHP, TsOH, CHCl_3_, 95%; (**b**) NaBH_4_, MeOH/Et_2_O = 1/1, quantitative yield; (**c**) NaH, CS_2_, CH_3_I, THF, reflux, 80%; (**d**) *o*-xylene, 190 °C, 4 h, 85%; (**e**) MsCl/Py, 0 °C, 73%; (**f**) AcOH, 50 °C, 95%; (**g**) 5% KOH (MeOH); (**h**) PCC/Al_2_O_3_, 80% from **16**; (**i**) H_2_/10% Pd(C), 95%; (**l**) CH_3_MgI/Et_2_O; (**m**) POCl_3_/Py.

The starting material for the obtaining of **8** was the ketol **12a** (vide supra). Thus **12a** was converted (Scheme 4) into the tetrahydropyranyl derivative **25** by standard methods. The latter was then reduced with NaBH_4_ to give the alcohol **26** which was transformed into the dithiocarbonate **27**. Pyrolysis of **27** gave then the unsaturated alcohol **28** which was converted into the corresponding mesylate **15**. The latter on heating in glacial AcOH underwent rearrangement to give **16** (see Section 8.2) which was converted into **8** by saponification followed by oxidation and catalytic hydrogenation [47]. Its structure was confirmed by its conversion into (+)-stemod-12-ene (**31**), identical with an authentic sample kindly donated by Dr. P. S. Manchand (Hoffmann-La Roche Inc., Nutley, NJ, USA).

The strategy adopted for the obtaining of tetracyclic ketones **7** and **8**, models of **5a** and **6** respectively, and the methodologies set-up during the model work, was so efficient that the following year formal total syntheses of (±)-**1** and (±)-**2a**, (±)-**2b** and total syntheses of (±)-**2c** and (±)-**2d** were achieved by a very small group of enthusiastic young people [13,48]. Later a diastereoselective total synthesis of (±)-**1** was also described [14].

## 3. Synthesis of (+)-Stemod-12-ene, (+)-2-Deoxystemodinone and (+)-Aphidicol-15-ene from (+)-Podocarpic Acid via (−)-9(11)-Podocarpen-12-one (1983–1984)

During the same time period Professor Ronald B. Kelly, a former co-worker of Professor Wiesner, got interested in the synthesis of aphidicolane, stemodane and stemarane diterpenoids and in 1980 reported with his group at the University of New Brunswick (Saint John, NB, Canada) the first total synthesis of (±)-stemarin (**3a**) [49]. In the following years the Kelly group focused its attention on stemodane and aphidicolane diterpenoids. In this frame the syntheses of (+)-stemod-12-ene (**31**), (+)-2-deoxystemodinone (**2d**) [50] and (+)-aphidicol-15-ene (**36**) [51] from (+)-podocarpic acid (**4**) were achieved.

### 3.1. Retrosynthetic Analysis and Strategy

**Scheme 5:**
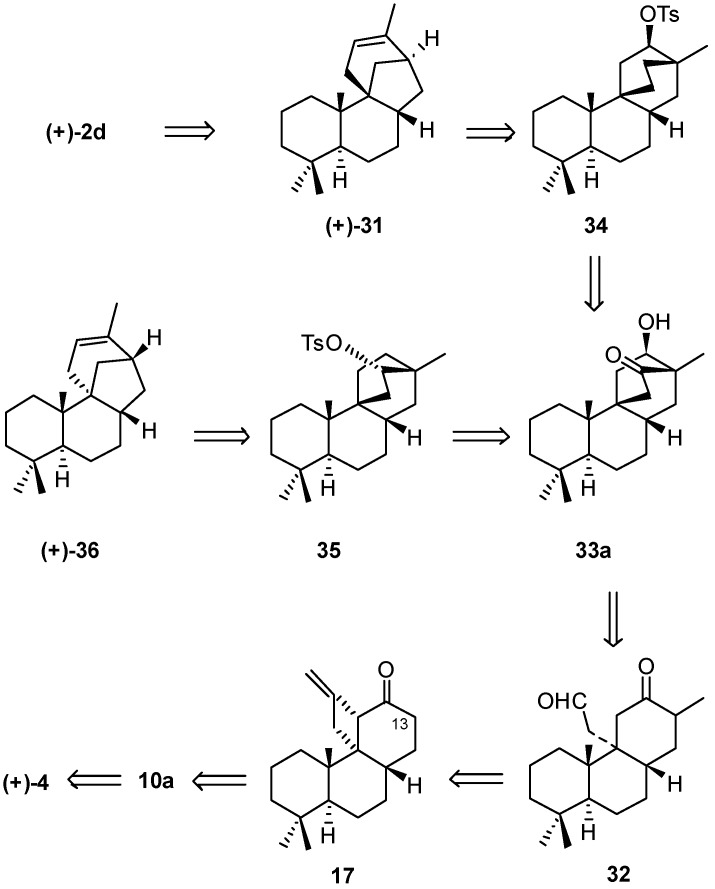
Retrosynthetic analysis for the obtaining of (+)-**31**, (+)**-2d** and (+)-**36**.

The approach adopted by Kelly and his group for obtaining (+)-stemod-12-ene (**31**), and (+)-aphidicol-15-ene (**36**) was also based on the bicyclo[2.2.2]octane → bicyclo[3.2.1]octane skeletal rearrangement (Scheme 5). (+)-Podocarpic acid (**4**) was also used as starting material. Being (+)-**31** and (+)-**36** the targets, a methyl group was introduced at C-13 in **17**. Its presence would later ensure the completeness of the rearrangement.

### 3.2. Synthesis of (+)-Stemod-12-ene and (+)-2-Deoxystemodinone (1983)

**Scheme 6:**
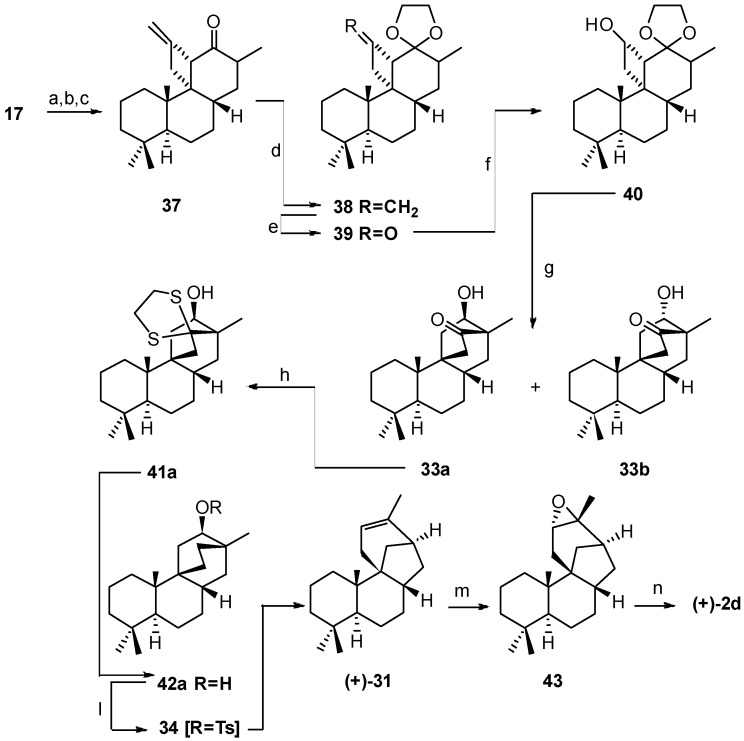
Synthesis of (+)-Stemod-12-ene and (+)-2-Deoxystemodinone. *Reagents and conditions*: (**a**) NaH, CO(OCH_3_)_2_, 97%; (**b**) NaH, MeI, 90%; (**c**) 10% KOH/EtOH, reflux, 89%; (**d**) ethylene glycol, TsOH, toluene, Dean-Stark, reflux, 87%; (**e**) OsO_4_, dioxane/Py/H_2_O/NaIO_4_, 77%; (**f**) NaBH_4_, EtOH, Et_2_O, 99%; (**g**) 1 N HCl, THF, reflux, 76%; (**h**) 1,2-ethanedithiol, BF_3_·Et_2_O, 0 °C, 85%; (**i**) Raney-Ni/EtOH, 83%; (**l**) TsCl/Py, 70%; (**m**) *m*-chloroperbenzoic acid, benzene, 75%; (**n**) LiAlH_4_, Et_2_O, 75%.

The starting material was the photoadduct **17** [47] (vide supra). Methylation at C(13) of the latter gave **37** which was acetalized to **38** by standard methods (Scheme 6). The exocyclic methylene in **38** was then cleaved to the cyclobutanone **39** and the latter reduced to the cyclobutanol **40** which on refluxing in a THF/HCl mixture underwent deacetalization followed by a retro-aldol and an aldol reaction to give **33** as an epimeric mixture at C(12) (see Section 8.1). The major epimer **33a** was then converted into the corresponding dithioacetal derivative **41a** and the latter desulphurized with Raney-Ni in EtOH to give **42a**. The alcohol **42a** was then transformed into the corresponding tosylate **34** which on heating in DMSO in the presence of the methyl sulfinyl carbanion gave (+)-stemod-12-ene (**31**) (see Section 8.3). (+)-2-Deoxystemodinone (**2d**) was eventually obtained from (+)-**31** by epoxidation followed by hydride epoxide reduction [50]. The use of (+)-podocarpic acid (**4**) as starting material also allowed confirmation of the absolute configuration attributed to the stemodane diterpenoids on the basis of ORD and CD studies.

### 3.3. Synthesis of (+)-Aphidicol-15-ene (1984)

**Scheme 7:**
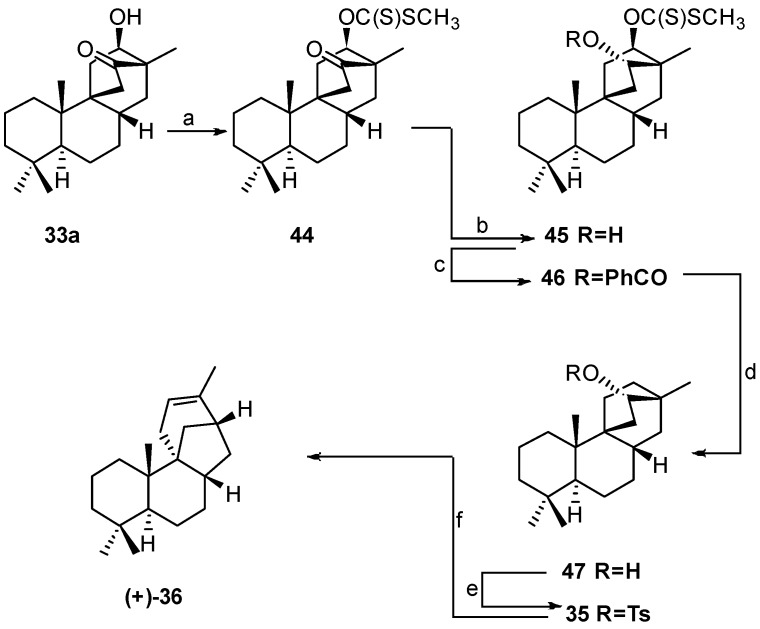
Synthesis of (+)-Aphidicol-15-ene. *Reagents and conditions*: (**a**) NaH, imidazole, THF, CS_2_, CH_3_I, 87%; (**b**) MeOH/Et_2_O = 1/1, NaBH_4_, −10 °C, 90%; (**c**) PhCOCl/Py, 97%; (**d**) Bu_3_SnH, toluene, reflux, 75%; (**e**) TsCl/Py, 3 °C, 72 h, 60%; (**f**) CH_3_S(O)CH_2_^−^Na^+^/CH_3_S(O)CH_3_, 60 °C, 2 h, 60%.

The starting material was **33a** (Scheme 7) which was converted into the dithiocarbonate **44**. The latter, upon reduction with NaBH_4_ gave diastereoselectively the alcohol **45** which was transformed into the benzoate **46**. Reduction of the latter with tri-*n*-butyltin hydride followed by saponification gave **47**. The conversion of **47** into (+)-**36** [51] paralleled that of **42a** into (+)-**31** [50] (see Section 8.4).

## 4. Proof of the Structure of the *Stemodia chilensis* Tetracyclic Diterpenoid (+)-19-Acetoxy-stemodan-12-ol by Synthesis from (+)-Podocarpic Acid (2016)

The aim of this work was to confirm the structure of (+)-19-acetoxystemodan-12-ol (**2f**) isolated in 1991 from *Stemodia chilensis* [52]. The structure of this diterpenoid had been established only on the basis of ^1^H- and ^13^C-NMR spectroscopic data and on their similarity with those of co-occurring (+)-2-deoxystemodinone (**2d**). The starting material was compound (+)-**48**, obtained from (+)-podocarpic acid (**4**) as previously described (see Section 7). The strategy adopted is that described in Scheme 5. The HO-C(12) in (+)-**48** is properly oriented for rearrangement to the stemodane system. Nevertheless, prior to rearrangement it was necessary to remove the C(15) carbonyl group, which would have prevented the rearrangement to the stemodane system by inhibiting the formation of a positive charge on the adjacent bridgehead carbon, and to convert the HO-C(12) into a better leaving group after selective protection of the HO-C(19).

**Scheme 8:**
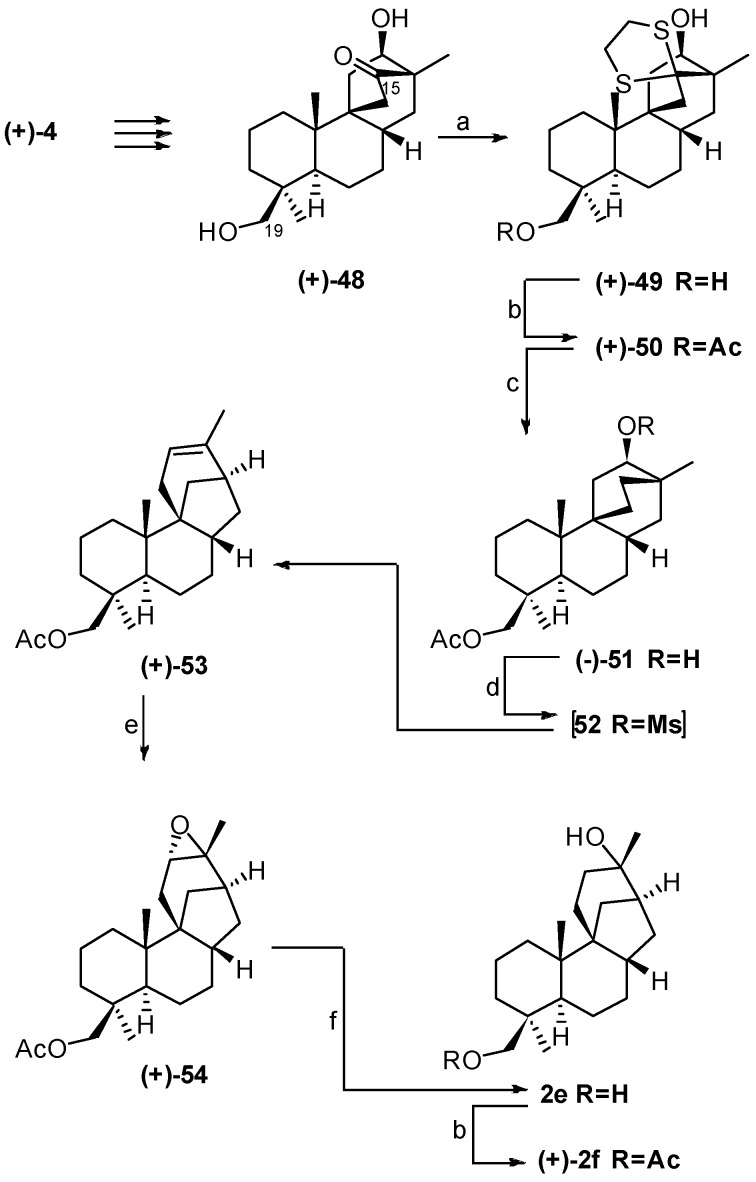
Synthesis of (+)-19-Acetoxy-stemodan-12-ol. *Reagents and conditions*: (**a**) 1,2-ethanedithiol, BF_3_·Et_2_O, 0 °C, 59%; (**b**) 2,6-lutidine, AcCl, CH_2_Cl_2_, rt, 83%; (**c**) Raney-Ni, EtOH, 60 °C, 80%; (**d**) MsCl, NEt_3_, CH_2_Cl_2_, rt, 66%; (**e**) *m*-chloroperbenzoic acid, CH_2_Cl_2_, 89%; (**f**) LiAlH_4_, Et_2_O, 40%.

Compound (+)-**48** (Scheme 8) was therefore converted by standard methods into the corresponding dithioacetal (+)-**49** which was chemoselectively acetylated to give (+)-**50**. Desulphurization of the latter gave (−)-**51**, which was treated with MsCl in pyridine to afford, via **52**, (+)-19-acetoxystemod-12-ene (**53**) (see Section 8.3). Compound (+)-**53**, whose relative configuration was confirmed by an X-ray structure determination, was then stereoselectively epoxidized to (+)-**54**. The epoxidation of the stemod-12-ene system proceeds, in fact, from the less hindered α face [50]. Reduction of (+)-**54** with LiAlH_4_ gave then the diol **2e** which was then acetylated to (+)-**2f** [53]. The identity of (+)-**2f** with the diterpenoid isolated from *Stemodia chilensis* was confirmed by comparing the NMR data. Remarkably in this synthesis all chiral centers present in (+)-podocarpic acid (**4**) are maintained.

## 5. Synthesis of (+)-Stemar-13-ene, (+)-18-Deoxystemarin from (+)-Podocarpic Acid via 9(11)-Podocarpen-12-one (1991–2012)

After the successful conclusion of the work on aphidicolane and stemodane diterpenoids, (±)-stemarin (**3a**) became the target of our studies. This tetracyclic diterpenoid had been synthesized in racemic form by Kelly and co-workers in 1980 [49]. Unfortunately this synthesis suffered a non-diastereoselective step, i.e., the completion of the synthesis with the minor epimer produced by the aldol condensation by which the bicyclo[2.2.2]octane system was formed. Developing a diastereoselective route to the key 6-hydroxy-1-methylbicyclo[2.2.2]octane intermediate appeared to us a worthwhile synthetic challenge. As it will be seen in the sequel this synthetic problem engaged us for twenty years until a simple solution was found. Being the synthetic problem confined in the construction of the C/D ring system, (+)-stemar-13-ene (**57**) and (+)-18-deoxystemarin (**3b**) the simplest compounds in the class were chosen as targets.

### 5.1. Retrosynthetic Analysis and Strategy

**Scheme 9:**
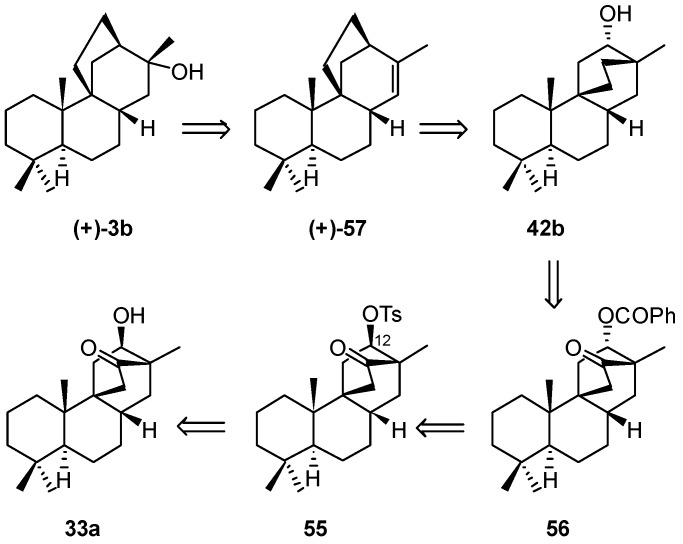
Retrosynthetic analysis for the obtaining of (+)-stemar-13-ene (**57**) and (+)-18-deoxystemarin (**3b**).

The first approach evaluated was based on the inversion of configuration at C(12) of suitable derivatives of the 13-methyl-12-endo-hydroxybicyclo[2.2.2]octan-15-one intermediate. Intermediates of type **55** were considered suitable to this task owing to the presence of the carbonyl group at C(15) which preventing the formation of a positive charge on the adjacent bridgehead carbon does not allow the rearrangement (Scheme 9).

### 5.2. Diastereoselective Synthesis of (+)-Stemar-13-ene and (+)-18-Deoxystemarin by Inversion of Configuration at C(12) of Suitable Derivatives of the 13-Methyl-12-endo-hydroxybicyclo[2.2.2]octan-15-one Intermediate (1991)

**Scheme 10:**
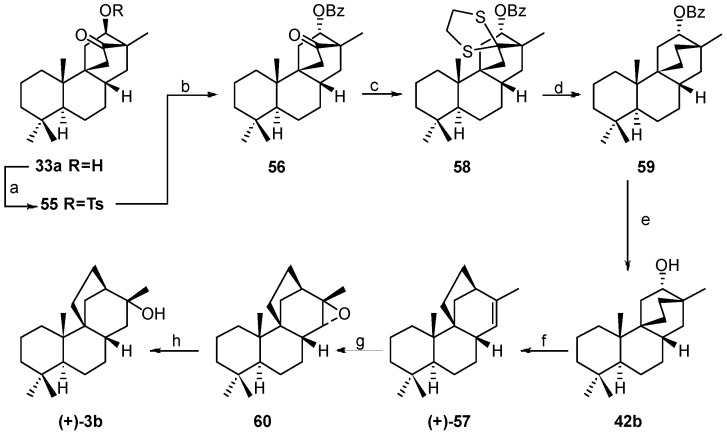
Synthesis of (+)-Stemar-13-ene and (+)-18-Deoxystemarin. *Reagents and conditions*: (**a**) TsCl/Py; (**b**) Et_4_N(PhCOO), CO(CH_3_)_2_, reflux; (**c**) 1,2-ethanedithiol, BF_3_·Et_2_O; (**d**) Raney-Ni, EtOH, reflux; (**e**) LiAlH_4_/THF; (**f**) TsOH, C_6_H_6_, reflux; (**g**) *m*-chloroperbenzoic acid, CH_2_Cl_2_; (**h**) LiAlH_4_, Et_2_O.

The starting material was known **33a** (vide supra) which was converted [54] into the corresponding tosylate **55** (Scheme 10). The latter was then treated with Et_4_N(PhCOO) in acetone at reflux affording the *exo*-benzoate **56**. Later the same conversion was performed in benzene resulting in drastically reduced reaction times [55]. This methodology, described in the past for the inversion of configuration of acyclic secondary alcohols [56], was particularly suitable to our case in that it produces a locked *exo*-ketol which cannot therefore re-equilibrate to the more stable *endo* epimer.

Deoxygenation of **56** via **58** by standard methods gave the benzoate **59**. Debenzoylation of the latter produced **42b** whose HO-C(12) is properly oriented for the rearrangement to the stemarane system. Thus refluxing the latter in benzene in the presence of TsOH gave (+)-stemar-13-ene (**57**) (see Section 8.5). Finally, epoxidation of the latter followed by LiAlH_4_ epoxide ring opening gave (+)-**3b** [54]. This synthesis was accomplished in 1991. In 2001 (+)-stemar-13-ene (**57**) was isolated from the fungus *Phoma betae* [57]. The two materials were identical in all respects.

Later, in view of a methodology [58] by which esters are cleaved in the presence CH_3_ONa/La(OTf)_3_ at neutral pH, thus ensuring no epimerization at HO-C(12), the desulphurization/debenzoylation steps could be reversed [59].

### 5.3. Synthesis of (+)-Stemar-13-ene and (+)-18-Deoxystemarin: Expeditious Preparation of the Key 13-Methyl-12-exo-hydroxybicyclo[2.2.2]octan-15-one Ethylene Dithioacetal (2008)

**Scheme 11:**
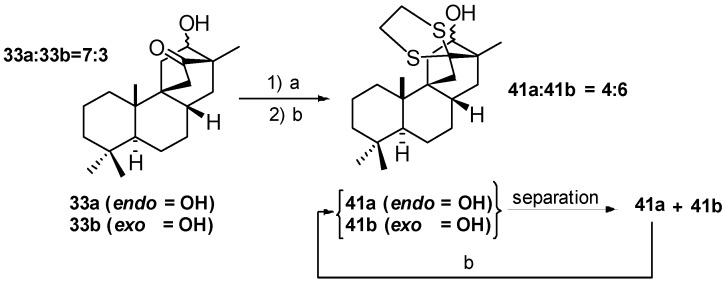
Equilibration under acidic condition of hydroxydithioacetals **41**. *Reagents and conditions*: (**a**) 1,2-ethanedithiol, BF_3_·Et_2_O, rt.; (**b**) TsOH, benzene, reflux.

Another approach considered for a diastereoselective route to the key 13-methyl-12-hydroxybicyclo[2.2.2]octanols of the type of **42b** concerned the possibility of obtaining ethylene dithioacetals of the type of **62** and **41b** by equilibration under acidic conditions (b in Scheme 11).

**Figure 3:**
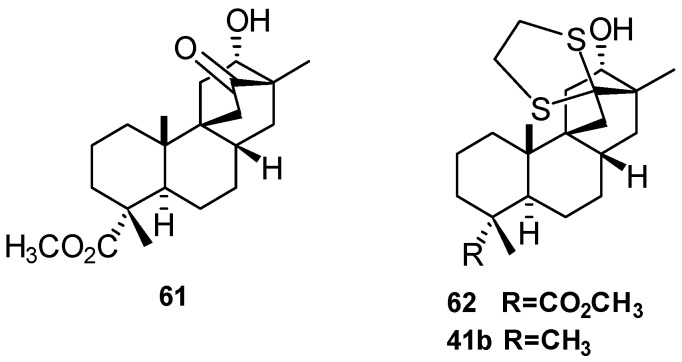
Key *exo*-Hydroxybicyclo[2.2.2]octane Ethylene Dithioacetals.

Compounds **62** and **41b** (Figure 3) can be efficiently prepared by thioacetalization with 1,2-ethanedithiol in the presence of BF_3_·Et_2_O from the corresponding 13-methyl-12-*exo*-hydroxybicyclo[2.2.2]octan-15-ones **61** and **33b** respectively.

Thus a 7:3 *endo*/*exo* mixture of hydroxydithioacetals **41**, prepared from **33** by the action of 1,2-ethanedithiol in the presence of BF_3_ Et_2_O, was submitted to equilibration with TsOH in benzene. The equilibrium *endo*/*exo* ratio resulted 4:6. Hydroxydithioacetals **41a** and **41b** could be separated by HPLC. By repeating three times the equilibration/separation cycle, practically all the material (94%) could be converted into the desired *exo* epimer **41b** [60].

## 6. Regio- and Diastereoselective Synthesis of (+)-Stemar-13-ene and (+)-18-Deoxystemarin by the 6-Hydroxy-1-methylbicyclo[2.2.2]octan-2-one → 4-Methylbicyclo[3.2.1]oct-3-en-6-one Skeletal Rearrangement (2011)

### 6.1. Retrosynthetic Analysis and Strategy

**Scheme 12:**
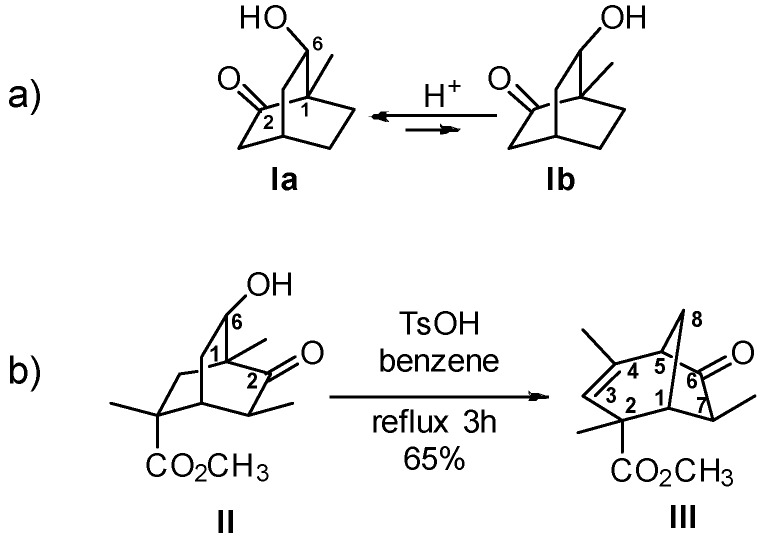
(**a**) *endo/exo* 1-methyl-6-hydroxybicyclo[2.2.2]octan-2-ones equilibrium under acidic conditions; (**b**) TsOH promoted rearrangement of 1-methyl-6-hydroxybicyclo[2.2.2]octan-2-ones to 4-methyl-bicyclo[3.2.1]oct-3-en-6-ones.

Finally, a new, general and simple solution for the construction of the stemarane diterpene C/D ring system was found. This solution is based on the existing *endo/exo* equilibrium under acidic conditions between 1-methyl-6-hydroxybicyclo[2.2.2]octan-2-ones (Scheme 12a) and on the acid catalyzed rearrangement of 1-methyl-6-hydroxybicyclo[2.2.2]octan-2-ones to 4-methylbicyclo[3.2.1]oct-3-en-6-ones (Scheme 12b) described in 2005 within the framework of a study on the reactivity of isotwistanes [61].

**Scheme 13:**
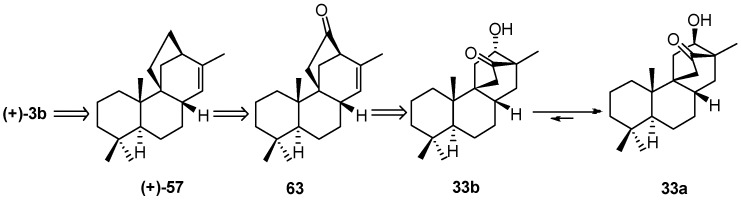
Retrosynthetic analysis for obtaining (+)-**57** and (+)-**3b** by TsOH promoted rearrangement of **33b**.

The following retrosynthetic analysis therefore resulted (Scheme 13).

### 6.2. Synthesis of (+)-Stemar-13-ene

**Scheme 14:**
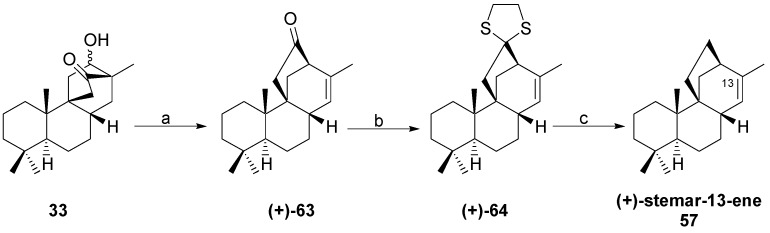
Synthesis of (+)-Stemar-13-ene (**57**) by TsOH promoted rearrangement of **33**. *Reagents and conditions*: (**a**) TsOH, toluene, reflux; (**b**) 1,2-ethanedithiol, BF_3_·Et_2_O, 0 °C, 85%; (**c**) Raney-Ni, EtOH, 60 °C, 76%.

Thus the 85:15 *endo/exo* mixture **33**, was dissolved in toluene and heated at 85 °C in the presence of TsOH (Scheme 14). After 24 h the rearrangement of **33** to (+)**-63** was completed (see Section 8.6). Thioacetalization of (+)**-63** by standard procedures afforded then (+)**-64** which was desulphurized to (+)**-57** [55,62].

**Figure 4:**
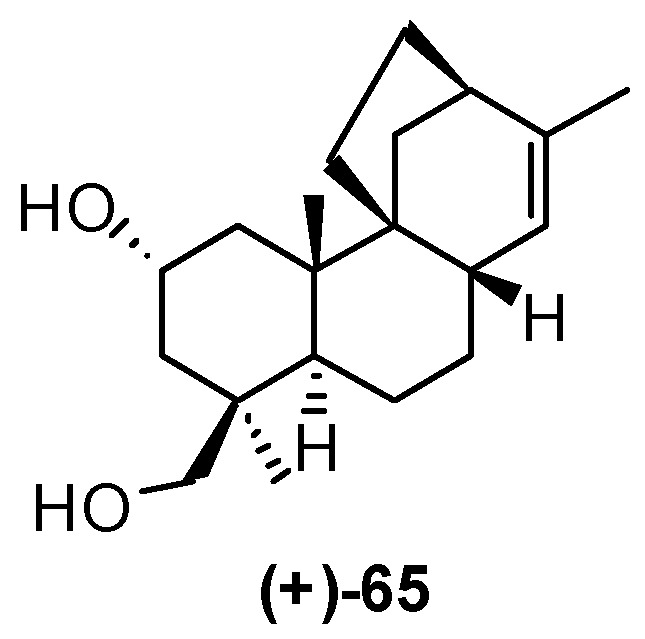
(+)-oryzalexin S.

The remarkable feature of this approach is that, owing to the stereospecificity of the rearrangement and to the 1-methyl-6-hydroxybicyclo[2.2.2]octan-2-ones *endo/exo* equilibrium, the whole *endo/exo* mixture **33** is converted into the rearrangement product in which the C(13)-C(14) double bond is also present, a characteristic feature of some stemarane diterpenoids such as (+)-oryzalexin S (**65)** (Figure 4) [63,64] (induced in rice leaves in response to the invasion of the fungus *Pyricularia oryzae* or when exposed to UV radiation or heavy metals [65]) and a necessary tool for the introduction of the α-configured HO-C(13) if necessary.

## 7. Regio- and Diastereoselective Synthesis of (+)-2-Deoxyoryzalexin S from (+)-Podocarpic Acid (2012)

**Scheme 15:**
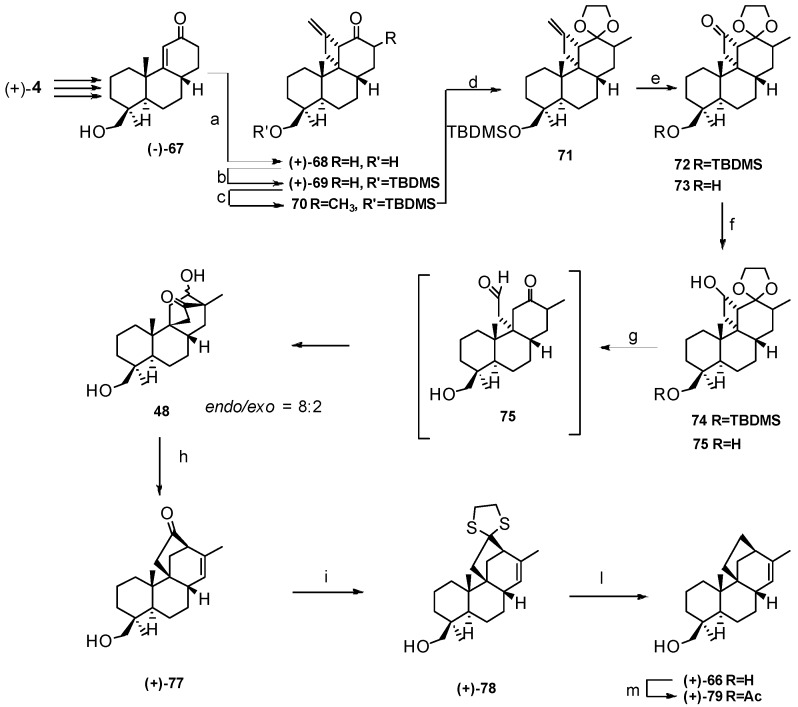
Regio- and Diastereoselective Synthesis of (+)-2-Deoxyoryzalexin S*. Reagents and conditions*: (**a**) allene, THF, −78 °C, hν, 81%; (**b**) TBDMSCl, imidazole, THF, 89%; (**c**) NaHDMS, CH_3_I, THF, −78 °C; (**d**) ethylene glycol, toluene, TsOH 78%; (**e**) OsO_4_/NaIO_4_, THF, H_2_O; (**f**) NaBH_4_, EtOH/MeOH, 0 °C; (**g**) THF/2N HCl, 88%; (**h**) TsOH, benzene, 85 °C, 66%; (**i**) 1,2-ethanedithiol, BF_3_·Et_2_O, 0 °C, 84%; (**l**) Raney-Ni, EtOH, 60 °C, 62%; (**m**) Ac_2_O/Py, 99%.

Finally, having in hand an efficient methodology for the construction of the C/D ring system of stemarane diterpenoids, we decided to apply it to the synthesis of (+)-2-deoxyoryzalexin S (**66**), the structure and absolute configuration of which had been established only on the basis of the corresponding ^1^H- and ^13^C-NMR spectra [66,67,68]. The strategy adopted is that described in Scheme 13. The starting material was (−)-19-hydroxypodocarp-9-en-12-one (**67**), available in four steps from (+)-podocarpic acid (**4**). Photoaddition of allene to (−)-**67** in THF at −78 °C gave quantitatively the photoadduct (+)-**68** the structure of which was established by 2D NMR experiments (Scheme 15). Prior to methylation at C(13), the HO-C(19) in (+)-**68** was protected to give (+)-**69**. Compound (+)-**69** was then methylated to give **70**. The latter was then converted into the acetal **71**, and the exocyclic methylene cleaved with OsO_4_/NaIO_4_ to give the cyclobutanone **72** along with some unprotected keto-alcohol **73**. Compound **72** was then reduced to the cyclobutanol **74**. Treatment of **74** with a 2:1 THF/2N HCl mixture gave **48** as an about 80:20 *endo/exo* C(12) epimeric mixture (see Section 8.1). The latter dissolved in toluene was heated at 85 °C in the presence of TsOH, giving (+)-**77** (see Section 8.6). Thioacetalization of (+)-**77** by standard methods followed by deoxygenation gave then (+)-2-deoxyoryzalexin S (**66)** which was then acetylated to (+)-**79** [69].

The relative configuration of (+)**-79** was confirmed by an X-ray structure determination. This work allowed also to demonstrate that the structure of (+)-2-deoxyoryzalexin S (**66**) could be not attributed to a Chilean *Calceolaria* isolated diterpenoid to which this structure had been assigned. Remarkably also in this synthesis all chiral centers present in (+)-podocarpic acid (**4**) are maintained.

## 8. Main Skeletal Rearrangements: Mechanistic Aspects

### 8.1. Conversion of Cyclobutanols ***19**, **40**, **74*** (and ***75***) into Hydroxybicyclo[2.2.2]octanones ***12**, **33***, and ***48**,* Respectively

**Scheme 16:**
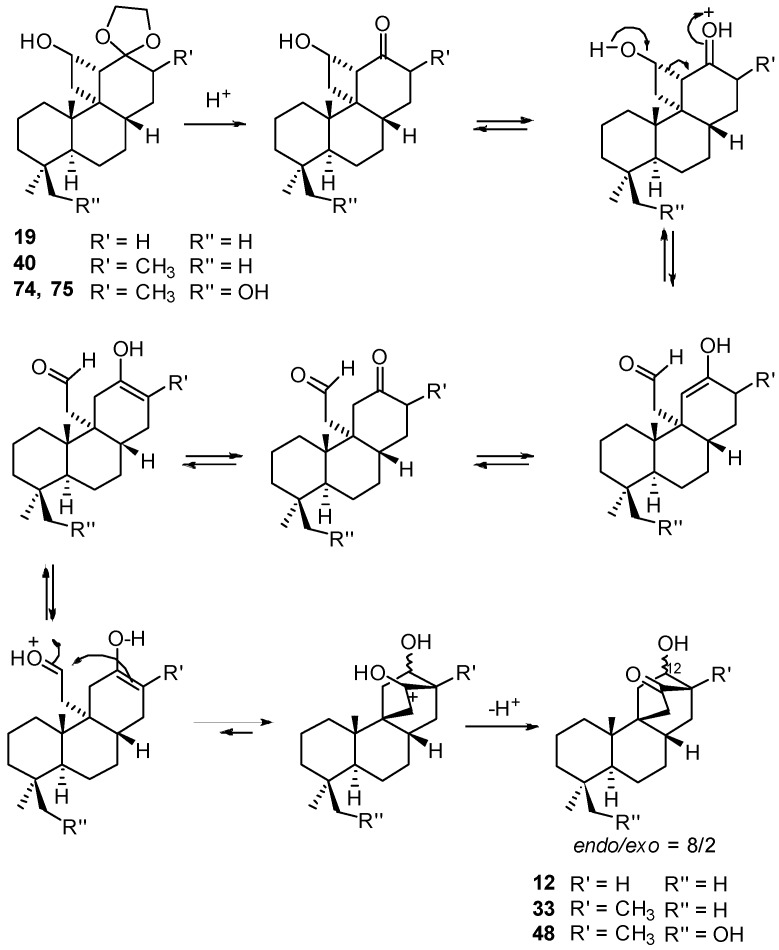
Conversion of Cyclobutanols **19**, **40**, **74** (and **75**) into Hydroxybicyclo[2.2.2]octanones **12**, **33**, and **48**, respectively.

Deprotection of the carbonyl function under acidic conditions unmasks a 1,3-hydroxyketone function which undergoes first a retro-aldol reaction then a new aldol reaction leading to the products (Scheme 16).

### 8.2. Skeletal Rearrangements of Mesylates ***13*** and ***15*** into Allylic Acetates ***14*** and ***16**,* Respectively

**Scheme 17:**
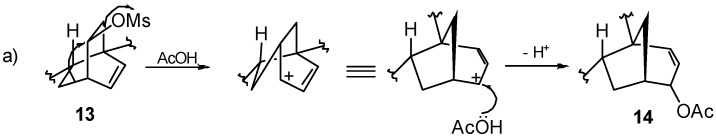

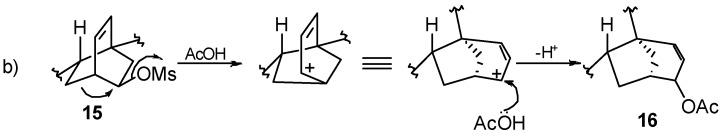
Skeletal Rearrangements of Mesylates **13** (**a**) and **15** (**b**) into Allylic Acetates **14** and **16**, respectively.

The presence of a double bond *endo* to the leaving group ensures a quantitative rearrangement by stabilizing the positive charge on the former bridgehead carbon (Scheme 17).

### 8.3. Skeletal Rearrangements of Compounds ***34*** and ***52*** into ***31*** and ***53**,* Respectively

**Scheme 18:**
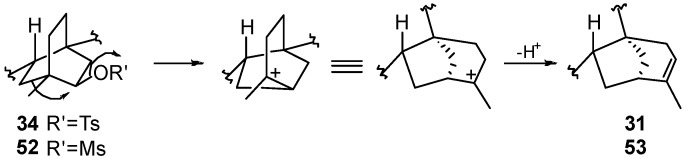
Skeletal Rearrangements of Compounds **34** and **52** into **31** and **53**, respectively.

The presence of a methyl group on the bridgehead carbon ensures a quantitative rearrangement by stabilizing the positive charge on the former bridgehead carbon (Scheme 18).

### 8.4. Skeletal Rearrangement of Tosylate ***35*** into ***36***

**Scheme 19:**
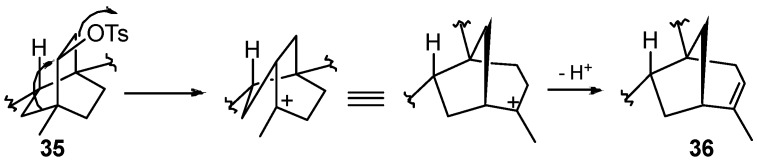
Skeletal Rearrangement of Tosylate **35** into **36**.

The presence of a methyl group on the bridgehead carbon ensures a quantitative rearrangement by stabilizing the positive charge on the former bridgehead carbon (Scheme 19).

### 8.5. Skeletal Rearrangement of ***42b*** into ***57***

**Scheme 20:**
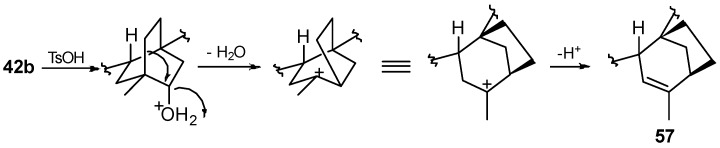
Skeletal Rearrangement of **42b** into **57**.

The presence of a methyl group on the bridgehead carbon ensures a quantitative rearrangement by stabilizing the positive charge on the former bridgehead carbon (Scheme 20).

### 8.6. Skeletal Rearrangements of ***33*** and ***48*** into ***63*** and ***77**,* Respectively

**Scheme 21:**
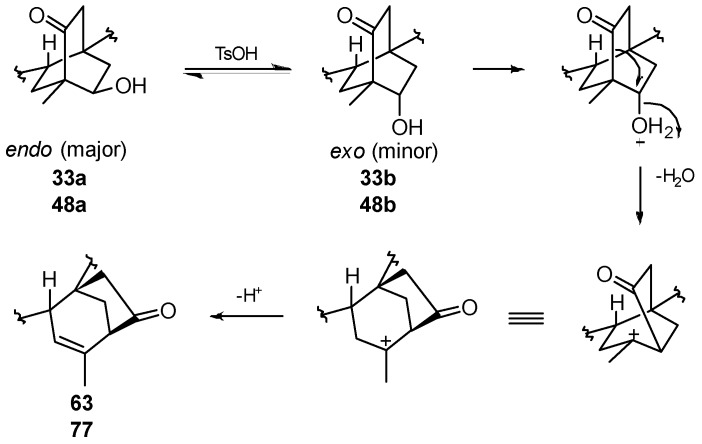
Skeletal Rearrangements of **33** and **48** into **63** and **77**, respectively.

The presence of a methyl group on the bridgehead carbon ensures a quantitative rearrangement by stabilizing the positive charge on the former bridgehead carbon (Scheme 21).

**Scheme 22:**
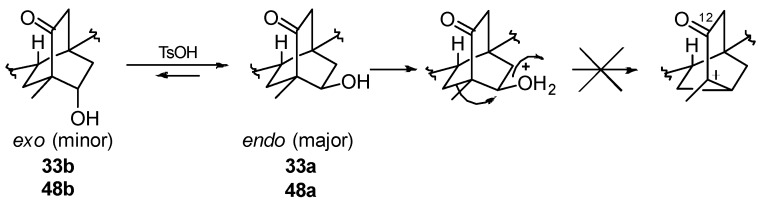
Role of the carbonyl in preventing the rearrangement of **33a** and **48a**.

The alternative rearrangement cannot occur owing to the presence of the carbonyl group at C(12) which prevents the formation of a positive charge on the adjacent bridgehead carbon (Scheme 22).

## 9. Conclusions

In this paper the use of (+)-podocarpic acid (**4**) as starting material for the synthesis of aphidicolane, stemodane and stemarane diterpenoids has been reviewed. As it could be seen (+)-podocarpic acid (**4**) was a very useful tool in a model study leading to the syntheses of tetracyclic ketones **7** and **8**, models of aphidicolin and stemodin syntheses key intermediates **5a** and **6** respectively [47]. (+)-Podocarpic acid (**4**) was then used for obtaining (+)-stemod-12-ene (**31**) and (+)-2-deoxystemodinone (**2d**), thus confirming the absolute configuration attributed to the stemodane diterpenoids on the basis of ORD and CD studies [50]. (+)-Podocarpic acid (**4**) was also converted into (+)-aphidicol-15-ene (**36**) [51] and into the *Stemodia chilensis* tetracyclic diterpenoid (+)-19-acetoxystemodan-12-ol (**2f**) allowing confirmation of the structure attributed to the latter only on the basis of NMR experiments [54]. (+)-Podocarpic acid (**4**) was then extensively used in the work which led to the synthesis of (+)-stemar-13-ene (**57**) and (+)-18-deoxystemarin (**3b**) [55,56,59,60,62]. (+)-Podocarpic acid (**4**) was also converted into (+)-2-deoxyoryzalexin S (**66**) [69]. The latter work also allowed us to demonstrate that the structure of (+)-2-deoxyoryzalexin S (**66**) cannot be attributed to a Chilean *Calceolaria* isolated diterpenoid. Finally, in view of its chemical transformations [70], (+)-podocarpic acid might represent a valuable starting material for the synthesis of (+)-aphidicolin (**1**), (+)-stemodin (**2a**) and (+)-stemarin (**3a**) analogues.
